# A rapid biosensor-based method for quantification of free and glucose-conjugated salicylic acid

**DOI:** 10.1186/1746-4811-4-28

**Published:** 2008-12-31

**Authors:** Christopher T DeFraia, Eric A Schmelz, Zhonglin Mou

**Affiliations:** 1 Department of Microbiology and Cell Science, University of Florida, P.O. Box 110700, Gainesville, FL, 32611, USA; 2 Center for Medical, Agricultural and Veterinary Entomology, United States Department of Agriculture, Agricultural Research Service, 1700 SW 23rd Drive, Gainesville, FL 32608, USA

## Abstract

**Background:**

Salicylic acid (SA) is an important signalling molecule in plant defenses against biotrophic pathogens. It is also involved in several other processes such as heat production, flowering, and germination. SA exists in the plant as free SA and as an inert glucose conjugate (salicylic acid 2-*O*-β-D-glucoside or SAG). Recently, Huang *et al*. developed a bacterial biosensor that responds to free SA but not SAG, designated as *Acinetobacter *sp. ADPWH_*lux*. In this paper we describe an improved methodology for *Acinetobacter *sp. ADPWH_*lux*-based free SA quantification, enabling high-throughput analysis, and present an approach for the quantification of SAG from crude plant extracts.

**Results:**

On the basis of the original biosensor-based method, we optimized extraction and quantification. SAG content was determined by treating crude extracts with β-glucosidase, then measuring the released free SA with the biosensor. β-glucosidase treatment released more SA in acetate buffer extract than in Luria-Bertani (LB) extract, while enzymatic hydrolysis in either solution released more free SA than acid hydrolysis. The biosensor-based method detected higher amounts of SA in pathogen-infected plants than did a GC/MS-based method. SA quantification of control and pathogen-treated wild-type and *sid2 *(SA induction-deficient) plants demonstrated the efficacy of the method described. Using the methods detailed here, we were able to detect as little as 0.28 μg SA/g FW. Samples typically had a standard deviation of up to 25% of the mean.

**Conclusion:**

The ability of *Acinetobacter *sp. ADPWH_*lux *to detect SA in a complex mixture, combined with the enzymatic hydrolysis of SAG in crude extract, allowed the development of a simple, rapid, and inexpensive method to simultaneously measure free and glucose-conjugated SA. This approach is amenable to a high-throughput format, which would further reduce the cost and time required for biosensor-based SA quantification. Possible applications of this approach include characterization of enzymes involved in SA metabolism, analysis of temporal changes in SA levels, and isolation of mutants with aberrant SA accumulation.

## Background

The plant signal molecule salicylic acid (SA) has been shown to play a role in several physiological processes, including heat production, flowering, germination and pathogen resistance [1-5]. In the last two decades, its role in pathogen resistance has been studied extensively [6,7]. Treatment with SA confers resistance to a variety of biotrophic pathogens [5,8], and pathogen infection causes the accumulation of SA [9,10]. SA can be glucosylated to form SAG (2-*O*-β-D-glucosylsalicylic acid), which serves as a biologically inert reservoir of SA [11]. SA is also present in plants as methyl-salicylate, which can also be conjugated to glucose [12]. Generally, mutants with constitutively high SA levels are resistant to biotrophic pathogens, while those unable to accumulate SA are susceptible [13-24]. Thus, quantification of SA is routine in the study of plant immunity.

The most commonly used methods for measuring SA from plant tissue employ HPLC or GC/MS [25-27]. These techniques both involve extraction of SA in organic solvents and subsequent evaporation. SA is then purified chromatographically, and detected by fluorescence spectroscopy or mass spectrometry. However, during extraction some of the SA is lost, and an internal control must be included to correct for SA recovery.

Recently, Huang *et al*. developed a biosensor for SA, named *Acinetobacter *sp. ADPWH_*lux *[28]. This strain is derived from *Acinetobacter *sp. ADP1, and contains a chromosomal integration of a salicylate-inducible *luxCDABE *operon, providing the substrate and catalyst for SA-responsive luminescence. The *Acinetobacter *sp. ADPWH_*lux *response appears to be limited to SA, methyl-SA, and the synthetic SA derivative acetylsalicylic acid [28]. Measurement of SA from TMV-infected tobacco leaves with the biosensor and GC/MS yielded similar results [29], demonstrating that this strain is suitable for the quantification of SA from plant tissue.

Herein, we present an improved approach for the quantification of free SA from Arabidopsis leaf extracts using *Acinetobacter *sp. ADPWH_*lux*. We have also developed a method for *Acinetobacter *sp. ADPWH_*lux*-based SAG measurement.

## Results

### Standard Curve Generation

**Figure 1:**
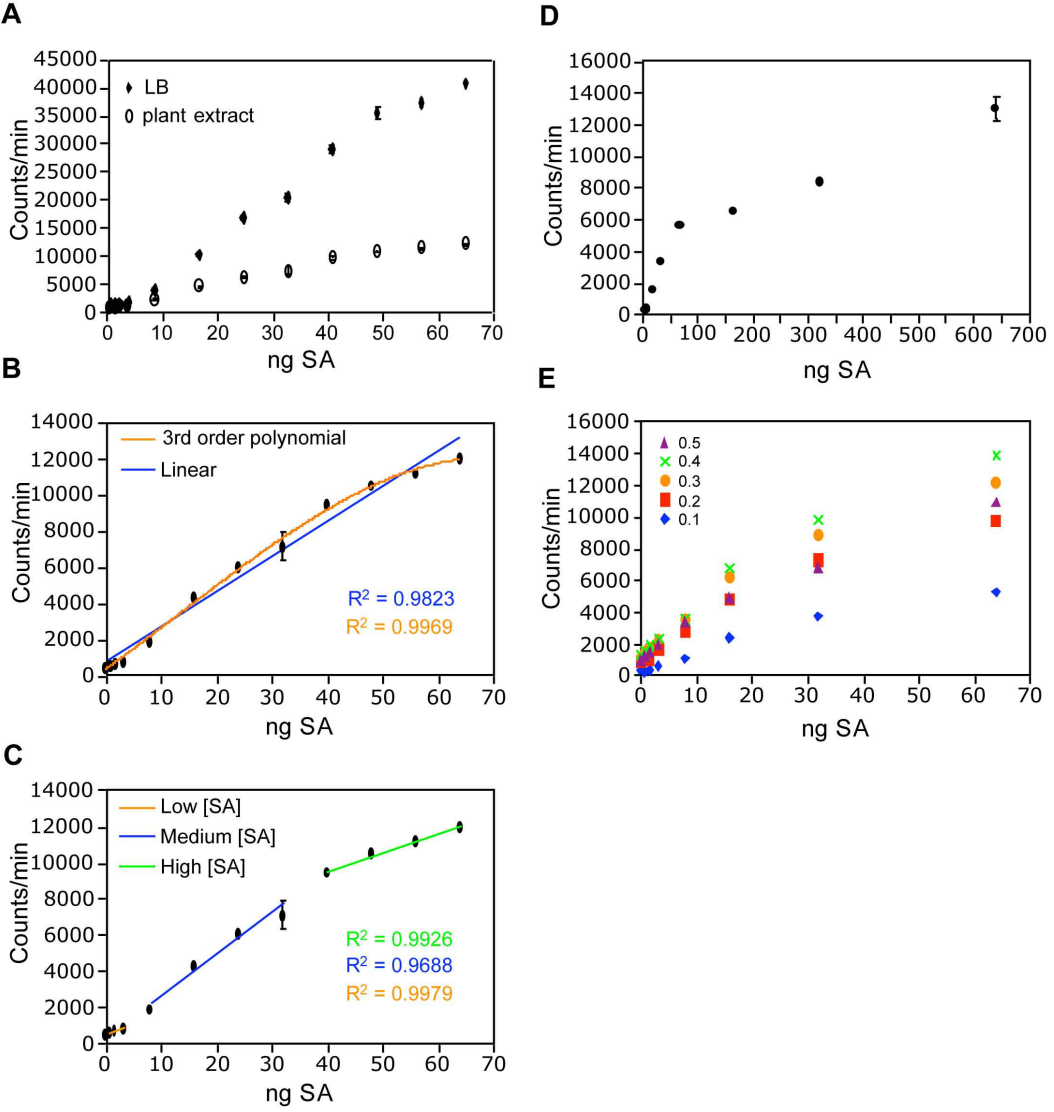
**Standard curve optimization**. (A) Effect of plant extract on SA-induced luminescence. SA standards were made with either LB or *sid2-2 *plant extract as the solvent. (B) Non-linearity of the SA-response curve. Data points were fitted with linear (blue) and third order polynomial (orange) best-fit lines. Note the lower R-squared value of the linear best-fit line. (C) A typical set of best-fit standard curves based on SA standards. The low SA concentration curve (orange) was fitted to standards of 0.8, 1.6, and 3.2 ng SA. The medium SA concentration curve (blue) was fitted to standards of 8, 16, 24, and 32 ng SA. The high SA concentration curve (green) was fitted to standards of 40, 48, 56, and 64 ng SA. (D) Diminishing response of the biosensor to increasing SA concentrations. (E) Effect of biosensor culture density on SA-induced luminescence. Biosensor cultures of OD_600 _= 0.6–0.8 were also tested and exhibited lower response to SA than OD_600 _= 0.4, but were omitted for clarity, as were error bars. Values indicate the average of three replicates with standard deviation (A-D only). Experiments were done three times with similar results.

Briefly, the method described by Huang *et al*. comprises tissue grinding, extraction in LB, sonication, and centrifugation, resulting in a crude plant extract containing SA. The crude extract is then mixed with a culture of the biosensor in a 96-well cell culture plate, and incubated at 37°C for one hour. The luminescence is then determined. In order to convert SA-induced luminescence into units of SA concentration, several standards with known amounts of SA are included to generate a standard curve [28]. We found that standards made with crude extract had significantly lower luminescence than those made with LB (Figure 1A), suggesting that the plant extract decreases induction of the biosensor by SA. Since our aim was to determine SA concentrations in plant extract, the standards must also have plant extract as a solvent. The ideal plant extract for making SA standards would initially contain no SA. In order to minimize the SA content of the extract used to make the standards, we used extract from *sid2-2 *plants, which fail to accumulate significant amounts of SA during pathogen infection. However, we and others [24] were unable to consistently detect a difference in constitutive SA levels between *sid2-2 *and wild type (data not shown). Therefore, untreated wild type plants may also be used for making the SA standards. Lack of a standard with no SA precludes the determination of absolute SA concentrations from plant extracts. Thus, the biosensor may only be used to determine relative SA levels between samples rather than absolute concentrations. When SA standards were made with plant extract, the relationship between luminescence and SA concentration was non-linear (Figure 1B). To simplify data analysis, instead of using all standards to construct the standard curve, only the standards with luminescence similar to that of the experimental sample were used. A best-fit linear line with a high R-squared value could then be derived and used as the standard curve (Figure 1C). Alternatively, a non-linear best-fit line can be used, although we found higher R-squared values for standards with low SA content, using the former method. Conversion from luminescence to SA concentration was carried out using the following equation:

[SA] = [(luminesence - y-intercept_standard curve_)/slope_standard curve_]/tissue mass

where known luminescence of a sample and tissue mass are used to calculate unknown SA concentration. In some cases, two or more standard curves were needed to determine the SA concentration of samples with largely different luminescence values. We found this approach to be useful in determining SA content between 1.6 and 64 ng SA (0.28 and 11 μg SA/g FW). At higher concentrations, induction of the biosensor by SA was diminished (Figure 1D). If sample SA concentrations exceeded 11 μg SA/g FW, the sample extract was diluted in untreated plant extract so that it fell within the useful range of the assay.

To determine if the culture density of the biosensor affected the useful range of the assay, we tested cultures of various optical densities (ODs) for SA-induced luminescence. The responsiveness of *Acinetobacter *sp. ADPWH_*lux *increased with culture density, reaching a maximum at OD_600 _= 0.4. Cultures with ODs higher than 0.4 were less responsive (Figure 1E), indicating that this is the optimum density for the assay. SA-induced luminescence varied somewhat between experiments (data not shown), so new SA standards were prepared for each experiment.

### Optimization of *Acinetobacter *sp. ADPWH_*lux*-based SA Measurement

**Figure 2:**
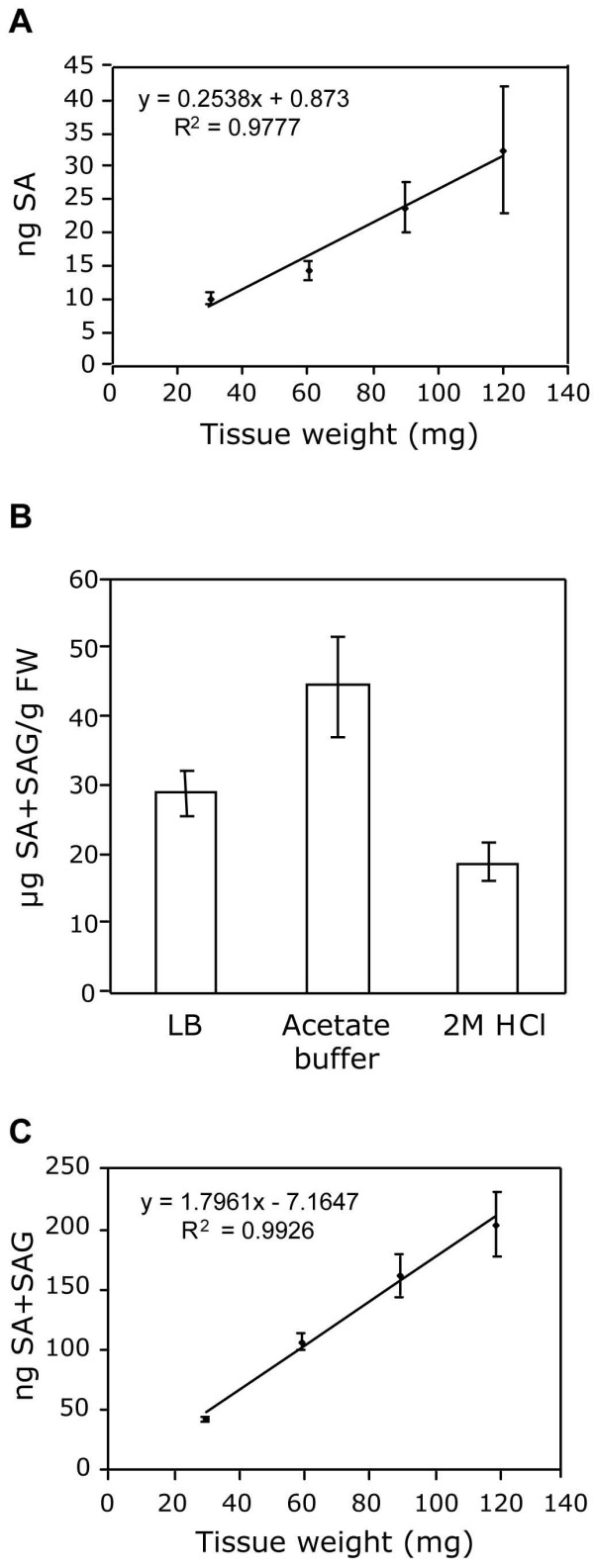
**Accuracy of ADPWH_lux-based SA and SAG quantification**. (A) SA measurement of varying *Psm *ES4326-infected tissue mass. (B) Comparison of extraction solvents for SA+SAG quantification. *Psm *ES4326-infected tissue was extracted with the indicated solvent. SA+SAG content was then determined as in Methods. (C) SA+SAG measurement of varying *Psm *ES4326-infected tissue mass. SA and SA+SAG measurements were done as described in Methods.

In order to further examine the specificity of the biosensor, we tested 12 substances similar in structure to SA, but not examined in [28] for their ability to induce luminescence in ADPWH_*lux*. These compounds are known to be present in plants, and/or accumulate during pathogen infection. None of the tested substances induced luminescence, even at high concentrations (Additional file 1). To improve upon the method of Huang *et al*. [28], a more rapid extraction protocol was tested. To extract many samples at once, we used a Genogrinder 2000 homogenizer to grind tissue that had been frozen in liquid nitrogen and to extract the samples in LB. Samples were centrifuged and the crude extract collected, omitting sonication. As described previously, the extract was mixed with biosensor culture and luminescence was measured [28]. SA content of wild-type plants infected with *Pseudomonas syringae *pv. *maculicola *(*Psm*) ES4326 measured by the modified method (described here) was similar to that obtained with the original method (3.50 ± 0.89 and 3.1 ± 0.73 μg SA/g FW, respectively), indicating that these changes did not significantly affect accuracy. To further confirm the accuracy of the assay, we measured SA from varying quantities of *Psm *ES4326-infected tissue. SA content increased linearly with tissue mass (R^2 ^= 0.9777, Figure 2A), confirming accuracy and suggesting little tissue (as few as 2–3 leaves) is needed to obtain reproducible results, allowing SA to be measured from single Arabidopsis plants without a completely destructive harvest. However, we typically used 5–6 leaves from different plants for each sample to minimize plant-to-plant variation.

### SAG Measurement

Although free SA is the biologically active form of SA, elevation of SAG concentration accompanies activation of plant defenses [30]. Consequently, measurement of SAG has been used for detecting alterations in SA metabolism [21]. Therefore, we developed a method for measuring SAG using the biosensor. SAG has previously been measured by treating a dried extract of SAG with β-glucosidase, releasing SA and glucose. The free SA is then analyzed by HPLC [15]. This involves several extraction steps, resulting in significant loss of SA. Since the biosensor detects SA in a complex mixture, we added β-glucosidase directly to the crude extract in order to avoid purification. Inclusion of β-glucosidase did not affect luminescence induced by free SA in a cell-free solution (Additional file 2). In the original biosensor-based protocol, SA was extracted in LB (pH 7.0). However, the optimum pH for β-glucosidase is 5.6 [31]. Enzymatic hydrolysis of purified SAG has been previously carried out in acetate buffer (0.1 M, pH 5.6) [30]. To determine whether LB or acetate buffer was better for β-glucosidase hydrolysis of SAG, we added β-glucosidase to crude extracts prepared with these two solutions. Additionally, we carried out acid hydrolysis of SAG [31]. Enzymatic hydrolysis of SAG in the acetate buffer extract released significantly more SA than in the LB extract (Figure 2B). An enzyme concentration of 0.03 U/ul crude extract was sufficient for maximum SAG hydrolysis for *Psm *ES4326-treated leaves (Additional file 3). Acid hydrolysis of SAG resulted in ~2-fold lower SA detection than enzymatic hydrolysis (Figure 2B); so acid hydrolysis was no longer employed. Free SA content from tissue extracted with acetate buffer did not differ significantly from tissue extracted with LB (data not shown). Thereafter, all crude extracts were prepared with acetate buffer, allowing the quantification of free and conjugated SA from a single sample. When SAG was measured in this way from varying quantities of *Psm *ES4326-infected tissue, SA+SAG content increased linearly with tissue mass (R^2 ^= 0.9926, Figure 2C).

### Comparison of ADPWH_*lux *and GC/MS Salicylic Acid Quantification

**Figure 3:**
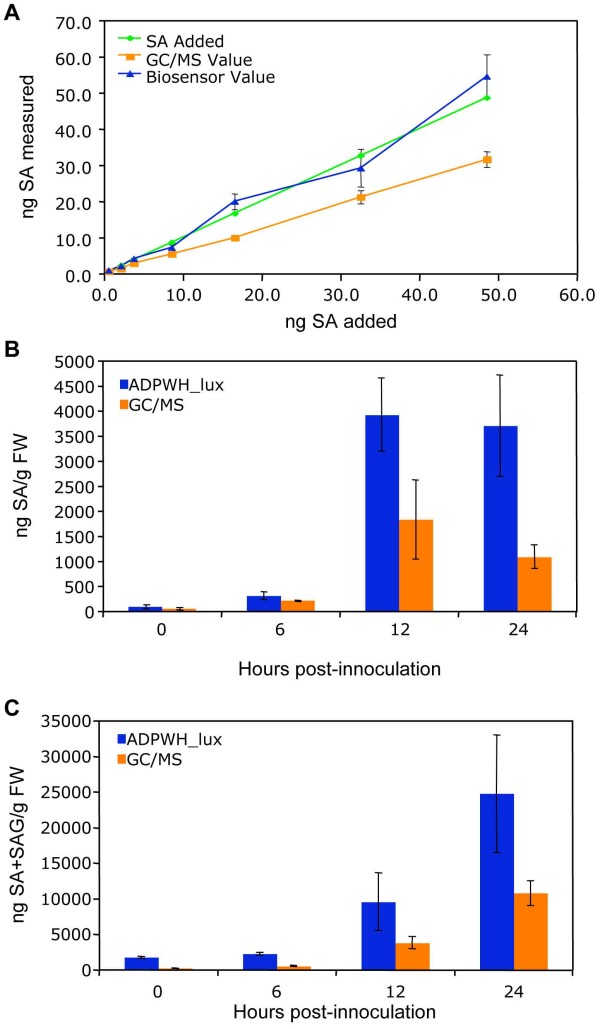
**Comparison of ADPWH_*lux*- and GC/MS-based methods for SA quantification**. (A) Quantification of SA from plant extracts with known amounts of SA added. The same extracts were used for SA quantification with each method. (B) Free SA from *Psm *ES4326-infected wild type. Known SA amounts added were 0.6, 2.2, 3.8, 8.6, 16.6, 32.6, and 48.6 ng. (C) SA+SAG from *Psm *ES4326-infected wild type. Values are the mean of 8 samples read in triplicate with standard deviation.

In order to compare our method of SA and SAG quantification with existing methods, we added known amounts of SA to plant extracts and analyzed them with ADPWH_*lux *and a previously established GC/MS method [27]. As shown in Figure 3A, the ADPWH_*lux*-based method detected higher levels of SA than did the GC/MS method, and the values reported by ADPWH_*lux *were closer to the amount of SA added. Both methods estimated values that increased linearly with increasing SA content. When the SA and SA+SAG content of *Psm *ES4326-infected wild type tissue was analyzed over time, the biosensor again reported higher concentrations than the GC/MS method. Both methods reported the highest concentration of free SA at 12 hpi, and the highest concentration of SA+SAG at 24 hpi (Figures 3A and 3B respectively).

### SA Accumulation in Wild Type and *sid2*

**Figure 4:**
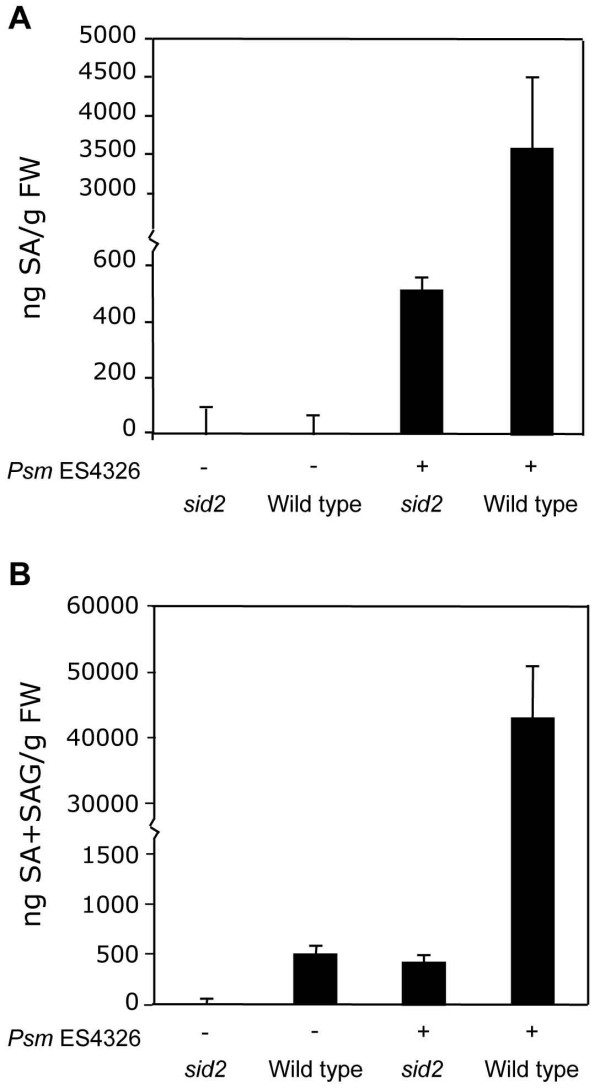
**SA measurement of untreated and *Psm *ES4326-infected wild-type and *sid2-2 *plants**. (A) SA. (B) SA+SAG. Values are the mean of 8 samples read in triplicate with standard deviation. Experiments were done three times with similar results.

To demonstrate the efficacy of ADPWH_*lux*, we measured SA and SAG in untreated and *Psm *ES4326-infected *sid2-2 *and wild-type plants. *Psm *ES4326 infection induced less SA and SAG accumulation in *sid2-2 *than in wild type (Figures 4A and 4B). After *Psm *ES4326 infection, in wild type, SA+SAG content was approximately 10-fold higher than SA content. This ratio is similar to those obtained in previous studies that used similar pathogen treatments [32-37] (Table 1). Wild type accumulated approximately six-fold more SA, and approximately 40-fold more SA+SAG than *sid2*. However, in wild type we obtained values for SA and SA+SAG that were significantly higher than those of previous studies (Table 1).

**Table 1:** Comparison of SA quantification results

Reference	Treatment	Dpi	Photoperiod (hr)	SA (μg/g FW)	SA+SAG (μg/g FW)
This Study	Untreated		16	ND	0.6

Lee *et al*., 2006 [32]	Untreated		16	0.1	0.5

Ishikawa *et al*., 2006 [33]	Untreated		12	0.3	0.8

Nandi *et al*., 2003 [34]	Untreated		14	-	.5

This Study	*Psm*ES4326 OD_600 _= 0.001	2	16	3.7	42

Zheng *et al*., 2007 [35]	*Psm*ES4326 OD_600 _= 0.0001	2	12	1.3	15

Gupta *et al*., 2000 [36]	*Psm*ES4326 OD_600 _= 0.002	1.5	12	0.6	5.6

Glazebrook *et al*., 2003 [37]	*Psm*ES4326 OD_600 _= 0.002	1.5	12	-	17

ND: Not detectable. A dash indicates SA was not determined.

### Evaluation of ADPWH_*lux*-based SA Quantification

The data presented in Figures 3 and 4 and in Table 1 suggest more SA may be detected using ADPWH_*lux *than with previous methods. One explanation is that the biosensor is responding to something other than SA that is present in the crude extract, resulting in artificially high values. Although several compounds that are structurally similar to SA and/or accumulate during the defense response do not induce luminescence in ADPWH_*lux *[28] (Additional file 1), we cannot exclude this possibility. Additionally, little luminescence was induced by pathogen-treated *sid2 *extracts, suggesting that if there is a compound other than SA that induces ADPWH_*lux*, it is not present in *sid2*, and may be derived from isochorismate. Another possibility is that recovery of SA using HPLC- and GC/MS-based methods which include organic solvent extraction and evaporation steps result in partial recovery of SA [38], despite inclusion of internal standards to account for the loss of SA. Although these internal standards have been shown to have similar recovery rates to SA [38], a difference in SA recovery between methods cannot be ruled out. Additionally, differences in photoperiod, pathogen inoculum, and the time after inoculation when SA content is measured, may also contribute to differences in SA measurements across different studies. Another possible cause of differing results across methodologies is methyl-SA accumulation, which induces luminescence in the biosensor [29]. However, in *Psm *ES4326-infected wild type, methyl-SA reached a maximum concentration of only 65 ng/g FW during pathogen infection (data not shown). Given this low value, it appears that methyl-SA accumulation contributes minimally to estimates of SA accumulation, and was therefore not included in the analysis.

**Figure 5:**
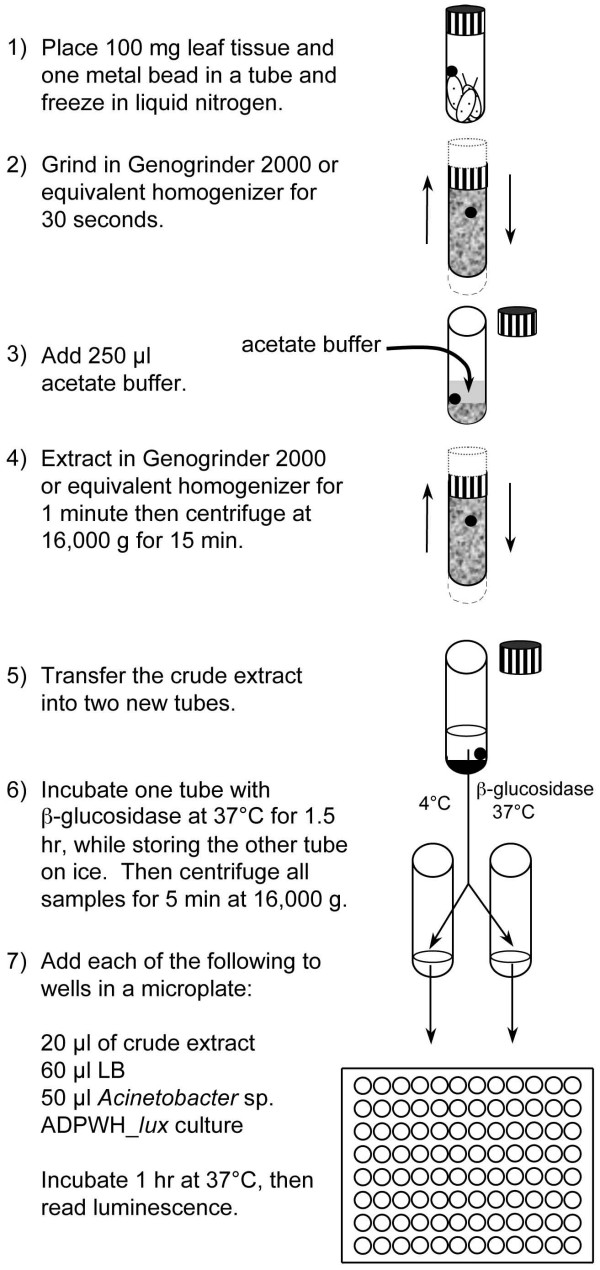
**Schematic of *Acinetobacter *sp. ADPWH_*lux*-based SA and SAG quantification**.

Despite differences with existing methods in terms of absolute SA concentration, the ADPWH_*lux*-based SA quantification is useful for comparing SA content in response to mutation and pathogen treatment. The values obtained for SA and SA+SAG was also highly reproducible. Consistently, free SA accumulation at 48 hpi was ~3.5 μg SA/g FW and SA + SAG was ~40 μg SA/g FW. The biosensor-based method routinely produced standard deviations between 15% and 25% of the mean and had a minimum detection limit of about 0.28 μg SA/g FW (data not shown). HPLC-based methods report standard deviations which are ~12% of the mean, and can vary in detection limit, depending on the protocol and instrumentation used [38,39]. A schematic of the biosensor-based methodology and a detailed protocol are presented in Figure 5 and Additional file 4, respectively. In our laboratory, free and conjugated SA was routinely quantified from ~50 samples in ~5.0 hr.

## Conclusion

In this study we present an improved method for the quantification of SA from plant tissue using the SA biosensor *Acinetobacter *sp. ADPWH_*lux*. The modified method is as accurate and more rapid than the previous *Acinetobacter *sp. ADPWH_*lux *-based approach [28]. We also developed a biosensor-based method for measuring SA + SAG using enzymatic hydrolysis. Free and conjugated SA can be measured simultaneously from hundreds of samples per day, providing an alternative to HPLC and GC/MS, with significant reductions in cost and processing time. Adoption of 96-well formats for tissue grinding, SA extraction, and SAG hydrolysis will further decrease the cost and time involved. It is our hope that this methodology will encourage investigators to include SA quantification in their experiments, facilitating a more thorough understanding of this intriguing molecule.

## Methods

### Preparation of crude extract

This procedure was adapted from Huang *et al*. [28]. SA measurements were carried out as follows unless otherwise indicated. On the day of SA measurement, samples were frozen in liquid nitrogen and ground at 1500 strokes/min for 30 sec in a Genogrinder 2000 (BT&C/OPS Diagnostics, Bridgewater, NJ). Tissue was ground three times while refreezing in liquid nitrogen each time. After the third round of grinding, samples were left at room temperature for 5 minutes, and 2.5 μl/mg tissue of room temperature acetate buffer (0.1 M, pH 5.6) was added. Samples were then mixed for 1 min at 1000 strokes/min and centrifuged for 15 min at 16,000 g. Half (100 μl) of the supernatant was stored on ice for free SA measurement and half was incubated at 37°C for 90 min with 4 U of β-glucosidase (3.2.1.21, Sigma-Aldrich, St. Louis, MO) for SAG measurement.

### Detection of salicylic acid using *Acinetobacter *sp. ADPWH_*lux *and GC/MS

An overnight culture of *Acinetobacter *sp. ADPWH_*lux *was diluted in 37°C LB (1:20) and grown for ~3 hrs at 200 rpm to an OD_600 _of 0.4. Twenty μl of room temperature crude extract was added to 60 μl room temperature LB in a black 96-well black cell culture plate. Using a multipipet, 50 μl of biosensor culture was added to each well and mixed by pipet action. The plate was incubated at 37°C for 1 hr without shaking before luminescence was read using a Victor3 Perkin Ellmer Multi-Detection Microplate Reader (PerkinElmer, Waltham, Massachusetts). Each sample was measured in triplicate. GC/MS based analysis of SA follows from Schmelz *et al*. [27]. Briefly, aliquots of crude extracts described above where spiked with 100 ng of ^2^H_6_-SA as an internal standard and mixed with 300 μl of H_2_O:1-propanol: HCl (1:2:0.005) followed by 1 ml of dichloromethane (MeCl_2_). The MeCl_2_:1-propanol layer containing SA was then transferred to a glass vial and 2 μl of 2.0 M trimethylsilyldiazomethane solution was added to form methyl esters. Residual derivatization agent was neutralized with excess acetic acid. Vapor phase extraction at 200°C was used to recover the MeSA on filters containing 30 mg Super Q (Alltech Associates, Inc., Deerfield, IL, USA) followed by elution with MeCl_2_. Samples were then analyzed with an established isobutane chemical ionization-GC/MS profiling method [27]. Estimates of salicylic acid (SA) represent combined pools of endogenous free acids and methyl esters.

### Standard curve

Known amounts of SA were dissolved in either LB or acetate buffer, then diluted 10-fold in plant extract. SA standards were read in parallel with the experimental samples. Conversion of luminescence to SA concentration was done as discussed in Results.

## Competing interests

The authors declare that they have no competing interests.

## Authors' contributions

CTD contributed to the conception and design of the project, collected, analyzed, and interpreted the data for all biosensor-based SA measurements, and prepared the manuscript. EAS collected, analyzed, and interpreted the data for all GC/MS-based SA measurements, and revised and edited the manuscript. ZM was involved in the conception and design of the project, revised and edited the manuscript, and is the PI of the laboratory. All authors read and approved the final manuscript.

## Supplementary Material

Additional file 1 **Specificity of ADPWH_*lux*.** The indicated compounds were added to ADPWH_*lux *and luminescence determined as described in Methods. Values are the mean of 4 samples read in triplicate with standard deviation. This experiment was done twice with similar results. Click here for file

Additional file 2 **Effect of β-glucosidase on free SA detection by ADPWH_*lux*.** β-glucosidase was added to plant extract containing known amounts of SA, and luminescence was determined with ADPWH_*lux *as described in Methods. Values are the mean of 4 samples read in triplicate with standard deviation. This experiment was done twice with similar results. Click here for file

Additional file 3 **Determination of the minimum effective quantity of β-glucosidase for the determination of SA+SAG.** β-glucosidase was added to *Psm *ES4326-treated plant extract in increasing amounts and SA+SAG was determined as described in Methods. Click here for file

Additional file 4 **Protocol.** Detailed protocol for *Acinetobacter *sp. ADPWH_*lux*-based SA measurement. Click here for file
