# Supplementary figures and images for: A rapid biosensor-based method for quantification of free and glucose-conjugated salicylic acid
Source: Plant Methods. 2008 Dec 31;4:28. doi: 10.1186/1746-4811-4-28 (PMC2654556; doi:10.1186/1746-4811-4-28)

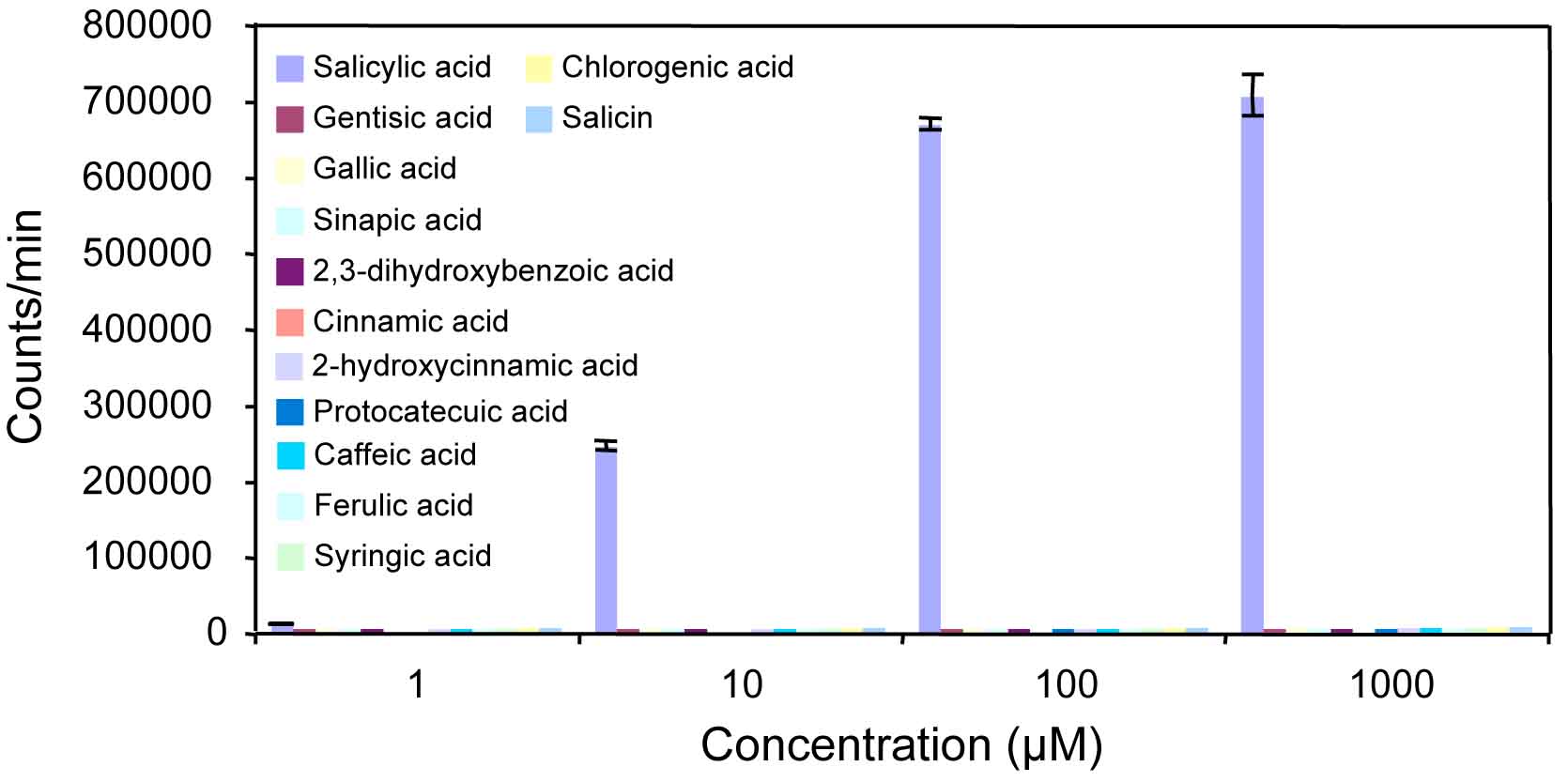

Supplement: Additional file 1 — Specificity of ADPWH_lux. The indicated compounds were added to ADPWH_lux and luminescence determined as described in Methods. Values are the mean of 4 samples read in triplicate with standard deviation. This experiment was done twice with similar results. [file 1746-4811-4-28-S1.jpeg]

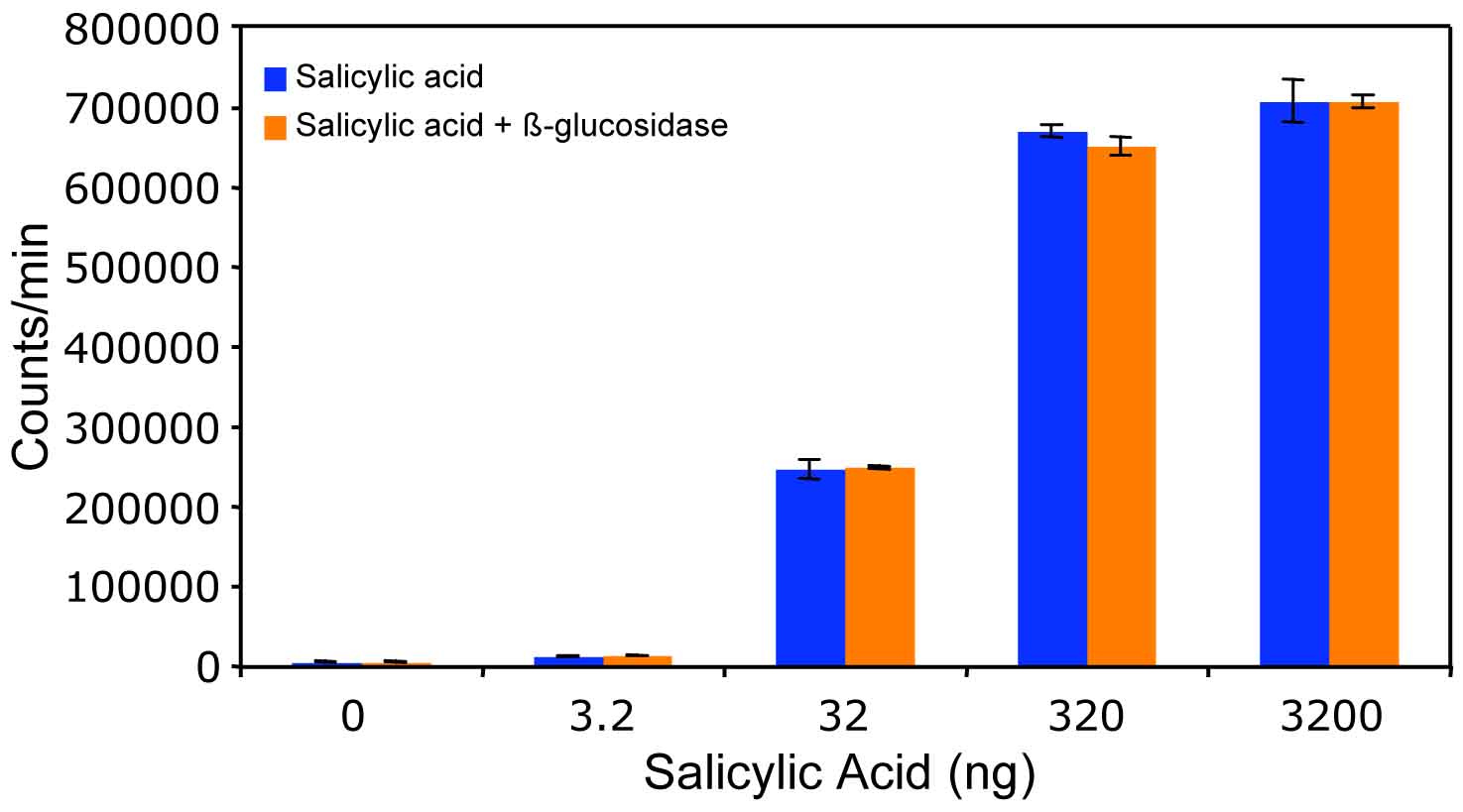

Supplement: Additional file 2 — Effect of β-glucosidase on free SA detection by ADPWH_lux. β-glucosidase was added to plant extract containing known amounts of SA, and luminescence was determined with ADPWH_lux as described in Methods. Values are the mean of 4 samples read in triplicate with standard deviation. This experiment was done twice with similar results. [file 1746-4811-4-28-S2.jpeg]

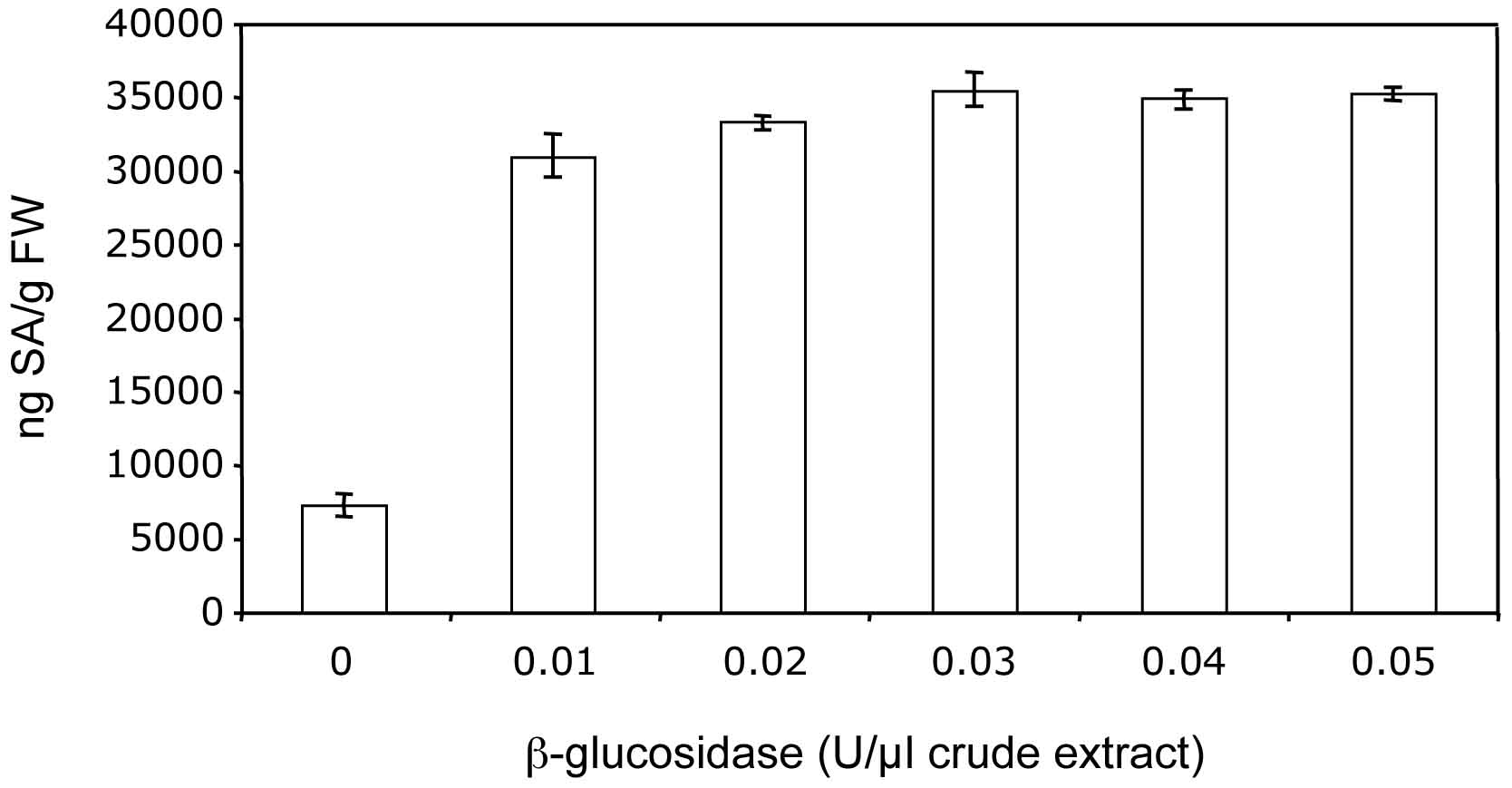

Supplement: Additional file 3 — Determination of the minimum effective quantity of β-glucosidase for the determination of SA+SAG. β-glucosidase was added to Psm ES4326-treated plant extract in increasing amounts and SA+SAG was determined as described in Methods. [file 1746-4811-4-28-S3.jpeg]
