# Supplementary material for: A rapid biosensor-based method for quantification of free and glucose-conjugated salicylic acid
Source: Plant Methods. 2008 Dec 31;4:28. doi: 10.1186/1746-4811-4-28 (PMC2654556; doi:10.1186/1746-4811-4-28)
Supplement: Additional file 4 — Protocol. Detailed protocol for Acinetobacter sp. ADPWH_lux-based SA measurement. [file 1746-4811-4-28-S4.doc]

Protocol for *Acinetobacter* sp. ADPWH_*lux*-based SA quantification

1. Harvest 100 mg leaf tissue and place into a 2 ml screw-cap tube and place a 4 mm steel bead (OPS Diagnostics, Lebanon, NJ, USA) in the tube. Freeze in liquid nitrogen and store at -80°C.
2. Freeze samples in liquid nitrogen. Set Genogrinder 2000 (or equivalent homogenizer) to 1500 strokes/min and grind samples for 30 seconds. Repeat grinding twice.
3. Add 250 μl 0.1M acetate buffer (pH 5.6) to the ground tissue, and mix in the genogrinder for 1 min at 1000 strokes/min.
4. Centrifuge for 15 min at 16,000 g at 4°C. Transfer 100 μl supernatant into each of two microfuge tubes (A PCR plate with a plastic cover can also be used if there are many samples), and then place one tube on ice for later free SA measurement.
5. Add 4 U of β-Glucosidase (3.2.1.21, Sigma-Aldrich, St. Louis, MO) to the supernatant, vortex, and incubate at 37°C for 90 min. During the incubation make the SA standards by diluting 10x stocks of SA solution in plant extract. Store standards on ice.
6. Add 60 μl LB to each well in a black 96-well cell culture plate. Add 20 μl of each plant (or less is the expected SA content is very high) extract and standard to each of three wells. If less volume of extract is used, an additional set of SA standards must be made using that volume of plant extract.
7. Add 50 μl of *Acinetobacter* sp. ADPWH_*lux* (OD_600_ = 0.4) to each well. Incubate the plate at 37°C for 1 hr.
8. Pre-warm the reader to 37°C and read luminescence.
